# Digitalisation Medical Records: Improving Efficiency and Reducing Burnout in Healthcare

**DOI:** 10.3390/ijerph20043441

**Published:** 2023-02-15

**Authors:** Nur Adibah Shaharul, Mohd ‘Ammar Ihsan Ahmad Zamzuri, Ahmad Azuhairi Ariffin, Ahmad Zaid Fattah Azman, Noor Khalili Mohd Ali

**Affiliations:** 1 Department of Community Health, Faculty of Medicine and Health Sciences, University Putra Malaysia, Serdang 43400, Malaysia; 2 Negeri Sembilan State Health Department, Ministry of Health Malaysia, Seremban 70300, Malaysia; 3 Seremban District Health Office, Ministry of Health Malaysia, Seremban 70590, Malaysia

**Keywords:** electronic medical record, healthcare system delivery, burnout, mental health, healthcare, information system

## Abstract

(1) Background: electronic medical record (EMR) systems remain a significant priority for the improvement of healthcare services. However, their implementation may have resulted in a burden on healthcare workers (HCWs). This study aimed to determine the prevalence of burnout symptoms among HCWs who use EMRs at their workplace, as well as burnout-associated factors. (2) Methods: an analytical cross-sectional study was conducted at six public health clinics equipped with an electronic medical record system. The respondents were from a heterogeneity of job descriptions. Consent was obtained before enrolment into the study. A questionnaire was distributed through an online platform. Ethical approval was secured. (3) Results: a total of 161 respondents were included in the final analysis, accounting for a 90.0% response rate. The prevalence of burnout symptoms was 10.7% (n = 17). Three significant predictors were obtained in the final model: experiencing ineffective screen layouts and navigation systems, experiencing physical or verbal abuse by patients, and having a poor relationship with colleagues. (4) Conclusions: the prevalence of burnout symptoms among healthcare workers working with electronic medical record systems was low. Despite several limitations and barriers to implementation, a paradigm shift is needed to equip all health sectors with electronic medical record systems to improve healthcare service delivery. Continuous technical support and financial resources are important to ensure a smooth transition and integration.

## 1. Introduction

Burnout is not categorised as a form of medical condition according to the International Classification of Disease (ICD-11). Burnout is often related to long-term workplace stress that is not adequately managed. It often refers to an occupational context, not related to other areas of experience in life [1]. The aetiology of burnout is multifactorial. It can be influenced by individual factors, occupational factors, personality factors, coping style, and other factors [2]. Critically, three dimensions of burnout are frequently explored by researchers: (i) feelings of energy depletion or exhaustion, (ii) increased mental distance from one’s job or feelings of negativity or cynicism about one’s job, and (iii) reduced professional efficacy [1].

Burnout conditions can occur in any occupation. This includes the medical fraternity, ranging across the span of a doctor’s life, affecting medical students, house officers, medical officers and specialists [3]. Based on a previous national report, an estimated 26.5% of Malaysia’s junior doctors experience burnout, particularly those who have worked for less than 6 months and those who are in emergency postings [4]. Furthermore, doctors in Malaysian urban hospitals are 5 times more likely to experience episodes of burnout (51%) compared to nurses, assistant medical officers, and hospital attendants [5]. Adding to these statistics, doctors working in a paediatric department in Malaysia are more likely to experience burnout compared to other departments such as accident and emergency, medical, orthopaedics, psychiatry, obstetrics and gynaecology, and surgery [5].

Previous research found that 74.5% (155/208) of the participants who reported burnout symptoms listed electronic medical record (EMR) systems as one of the contributing factors [3]. Recent qualitative research indicated that the use of EMR systems may have a detrimental effect on clinical reasoning and interprofessional collaborative practices [6]. However, EMRs have helped to improve doctors’ dedication to their work because they can assist clinicians in building the narrative of the patient [6]. Hence, achieving nationwide adoption of EMR systems remains a significant priority for the improvement of healthcare services. However, their implementation may have resulted in hospitals incurring expensive upfront and ongoing costs, experiencing difficulty in medical collaboration, and, ultimately, being unable to use the systems, in practice, due to limited resources [7].

In 1989, Davis explained, using the technology acceptance model (TAM), that acceptance can be influenced by external variables, perceived usefulness, perceived ease of use, attitude towards using, behavioural intention to use, and actual system use. The perceived usefulness of the system occurs when the product improves job performance or assists in accomplishing tasks quickly and efficiently. The perceived ease of use occurs when users believe the product is flexible and easy to understand, and when it is easy to become skilled at using it [8]. A mixed-methods study in Malaysia classified challenges in data completeness in hospital information systems into system factors and human factors [9]. Therefore, in the present study, besides sociodemographic and occupational factors, the EMR system’s intrinsic (related to the software and hardware) and extrinsic (related to human factors and overall satisfaction) factors were examined.

Significant challenges in adopting EMR system tools have been reported in many studies. A study in Malaysia found that issues related to EMR adoption were cost, technology, and human and legal-related concerns, and proposed an EMR adoption framework [10]. It reviewed the current situation of hospital information system (HIS) integration based on models such as the theory of reason action and the theory of planned behaviour, in addition to the TAM. That study then classified potential barriers and facilitators for using EMRs into three categories: organisational challenges, human challenges, and technological challenges.

In Malaysia, studies related to EMR systems have focused on their implementation in hospitals [11,12,13]. Exhaustive studies in similar settings have been performed abroad [14,15,16]. To our knowledge, limited studies have investigated EMR implementation in primary healthcare clinics (PHCs) or *Klinik Kesihatan* [17,18]. In the hospital EMR system, sometimes referred to as the HIS or clinical information system (CIS), a larger network of data management exists for hospital environments. The HIS is an important tool in improving a hospital’s operations and services. However, only 15.2% of Malaysian public hospitals have implemented a HIS. One of the known factors in the slow progress of adopting HIS is human context, with intertwining contributions of technological maturity, organisational preparedness, and environmental context [19].

The implementation of EMR systems creates both favourable and unfavourable outcomes for the end user [20]. One aspect is burnout symptoms among healthcare workers (HCWs), reflected by overall dissatisfaction and frustration with using the EMR system [20]. This is because any alteration to the existing workflow, such as replacing a manual medical records system with an EMR system, modifies the usual work processes in a PHC [21]. Despite the known advantages when the technology is utilised, the implementation of an EMR system in a PHC inevitably causes burnout symptoms. Hence, this study aimed to determine the factors contributing towards burnout among HCWs using EMR systems in PHCs and to measure its health burden. Ultimately, the findings of this study may contribute as a guide in the focus area of mental health burden among HCWs in PHCs and aid in planning appropriate interventions. The research questions for this study were: (1) What is the prevalence of burnout among HCWs using EMR systems in PHCs? (2) What factors contribute to burnout among HCWs using EMR systems in PHCs? (3) What are the predictors of burnout among HCWs using EMR systems in PHCs?

## 2. Materials and Methods

### 2.1. Study Setting

This was an analytical cross-sectional study conducted in Seremban District, Negeri Sembilan, Malaysia, from October 2020 to March 2021. Six PHCs were chosen for a pilot project in 2015 under the Seremban district health office to use an EMR system, named Tele Primary Care and Oral Health Clinic Information System (TPC-OHCIS), from 2017 [22]. The TPC-OHCIS system is a one-on-one end-user system that requires all job categories to have unique user IDs and computers (desktops or laptops) to access the system at the specific health facility. TPC-OHCIS is one of the e-Health initiatives of the Ministry of Health Malaysia. It is designed to integrate and improve the existing EMR system and be used separately in PHCs. Teleprimary Care (TPC) is used in PHCs, and the Oral Health Clinical Information System (OHCIS) is for dental clinics; both systems were integrated into TPC-OHCIS. The TPC-OHCIS system is currently used as the daily real-time operating system, for example, for data entry. Subsequently, the system has taken over the overall functions of other clinical systems in use. Thus, patient health records can now span information, from prenatal care to elderly care, in a ‘womb to tomb’ approach [22]. Hence, these six PHCs were chosen as the study setting. These PHCs are also among the busiest clinics in Seremban District and have a total of 755 HCW users of the TPC-OHCIS system.

### 2.2. Study Participants and Samples Size

The participants in this study were HCWs from different backgrounds: family medicine specialists (FMSs), medical officers (MOs), assistant MOs, and nurses. The exclusion criteria were HCWs currently diagnosed and receiving treatment for mental illnesses. The inclusion criteria were:

1. HCWs who use the TPC-OHCIS system, encompassing FMSs, MOs, assistant MOs, and nurses.
2. HCWs currently working in the six PHCs in Seremban District.
3. HCWs with at least one month of working experience using the EMR system in their PHC.

The sample size (n) in this study was calculated using the two proportions formulated by Lwanga and Lemeshow in 1990 [23]. The estimation of the sample size in this study was based on Siau et al. (2018) [5]. Based on this calculation, n = 65 HCWs for each group: doctors and nurses. Considering the adjustment for the comparison between the 2 groups, the total number of samples was 131 HCWs.

### 2.3. Study Instruments

An online, self-administered, structured questionnaire, consisting of five main sections, was used. The sections were (A) sociodemography, six items; (B) occupational factors, seven items; (C) EMR system intrinsic factors, seven items; (D) EMR system extrinsic factors, six items; and (E) adapted Mini Z survey to measure burnout.

Various models are used to explore the acceptance of EMR systems in healthcare industries. The most common are the TAM [1] and the unified theory of acceptance of use of technology (UTAUT) model [2]. Another simple method is to group EMR system implementation challenges into system and human factors [9]. System-related factors include old hardware and insufficient availability, stability in networks and connectivity, difficulty in retrieving data, the flexibility of the EMR system, and interoperability issues; these were referred to as intrinsic factors for Section C. Overcoming these issues strategically enables the prevention of user resistance towards using the EMR system and avoiding burnout.

For Section D, the EMR system’s extrinsic factors were defined as features—in addition to the built-in digital system—on the user’s and management’s side, that have an impact on the system implementation [3]. From the same model as the EMR system’s intrinsic factors, the TAM by Davis (1989) [1] explains these external factors that influence the acceptance of technology by the end user [8]. Another study also suggested that EMR system implementation challenges can be grouped into system and human factors [9]. Extrinsic factors, referred to as human-related factors, include the habit of entering data in free-text format, using copy/paste without verification, poor inter-personnel communication, user non-compliance with operating protocols, and user incompetence [9].

The original Mini Z instrument was developed from the physician work–life study [24], consisting of ten questions using a five-point Likert scale, and one open-ended question at the end. These ten items assess three outcomes: burnout, stress, and satisfaction. The seven drivers of burnout consist of four deriving from occupational factors—(i) work control, (ii) work chaos, (iii) teamwork, and (iv) value alignment with leadership—and three from EMR system components—(i) documentation time pressure, (ii) EMR use at home, and (iii) EMR proficiency. Of the three outcomes (burnout, stress, and satisfaction), stress was excluded from this study because it was not the outcome of interest, and satisfaction was repositioned into Section D—EMR system extrinsic factors. Of the seven drivers of burnout, two work-related items—work control and work chaos—were excluded during content validation by experts because they were not clear in the local context. Teamwork and value alignment with leadership were translated and retranslated and included in Section B: occupational factors. Only two out of three EMR system components in the original Mini Z were retained in this study because Item b, EMR use at home, did not relate to the local context since the system is only for use in healthcare facilities.

Some studies have only used a single question item (Q #2) to reflect burnout symptoms, rather than all items in the Mini Z questionnaire. This single-item tool has been demonstrated to be strongly correlated with the emotional exhaustion scale of the Maslach Burnout Inventory (MBI) [25]. The Pearson correlations and ANOVA used in the single-item measurement of burnout in the Mini Z against the 22-item MBI was r = 0.64 (*p* < 0.0001) with emotional exhaustion and R^2^ of 0.5 (*p* < 0.0001) [25]. Hence, this study opted to use a single-item questionnaire to measure the emotional exhaustion burnout component, given that it is an alternative to the MBI, with the ultimate intention of increasing the response rate [25]. Some of the items were adapted from the Mini Z questionnaire, some (using EMR at home) were not relevant to the local context, and a few new items were added regarding occupational factors and the EMR system’s application in the PHCs.

The content of the questionnaire was assessed by the members of the supervisory team and other panels of experts in the field, such as public health medicine specialists with experience working closely with the EMR system in PHCs, and HCWs who were involved in using TPC-OHCIS. All elements of components were measured, such as sociodemographic and occupational factors, the EMR system’s intrinsic and extrinsic factors, and burnout. Corrections and comments were made based on suggestions and recommendations. The content validity ratio (CVR) was used. Thus, the CVR value for all the main 26 items in the questionnaire was 1. Lawshe’s CVR method, introduced in 1975, is popular in scale development for health and education sciences. The CVR value is between 0 and 1 if the essential item is more than half but less than all, and it is negative when the expert-rated essential item is less than half [26]. The face validity of the English-language questionnaire was assessed during the pre-test of the questionnaire with five non-expert judges, who commented on its language, structure, and sentences. Due to minor adaptation of the items in the questionnaire, a new internal consistency reliability was estimated using Cronbach’s alpha (CA). Based on the pilot study performed with 43 HCWs who were EMR users in Kuala Lumpur and Putrajaya PHCs, the CA was 0.716 for occupational factors (3 items), 0.873 for the EMR system’s intrinsic factors (7 items) and 0.740 for the EMR system’s extrinsic factors (5 items). The overall CA for all 15 Likert-scale items was 0.902.

### 2.4. Data Collection

After Medical Research and Ethics Committee approval on 9 June 2021, and meticulous discussion with the Negeri Sembilan State Health Department, leading to approval on 15 June 2021, data collection for this study was conducted using the validated, structured questionnaire for a duration of one week (15 June 2021 to 22 June 2021). A name list of HCWs in PHC clinics using the EMR system was obtained from the responsible officer before data collection and randomization were performed. After permission was granted by the site supervisor in Seremban, the HCW was provided a Google form link. They were invited to participate in this study after the objectives of the study were explained to them, and after they were advised to read the information sheet provided online. The expected duration to answer the questionnaire was five to ten minutes for each respondent. The online patient information sheet was completed and informed consent was obtained if the HCW agreed to participate in the study. The confidentiality of each HCW was maintained throughout the process. Participants were assured that their identities would remain anonymous, and data would be further analysed to maintain the validity of the data. This study abided by the Declaration of Helsinki.

### 2.5. Data Analysis

Data analysis was carried out using the IBM Statistical Package for Social Sciences (SPSS) version 26.0. Descriptive analysis for continuous data was expressed as median and IQR, as all continuous data were not normally distributed. Descriptive analysis for categorical data was performed by using frequency and percentage. The dependent variable for this study was burnout symptom, measured using the adapted Mini-Z questions of burnouts. For bivariate analysis, categorical data were analysed using Chi-square or Fisher’s exact test if the requirements of the Chi-square test were not met. All categorical variables that were significant in the bivariate analysis were included in formulating multivariate analysis by using multiple logistic regressions (MLogR). A value of *p* < 0.05 was considered statically significant. The data collection was analysed by using an intention-to-treat basis.

### 2.6. Ethical Approval

The research was registered with National Medical Research Register (NMRR) with NMRR registration number NMRR-21-551-58900 (IIR) and received approval from the Medical Ethics Committee (MREC), Ministry of Health Malaysia. Written consent was acquired from participants before the study. Participants’ information from this study is kept strictly confidential and their anonymity was maintained. The documents for informed consent and the information sheet were distributed online to the participants.

## 3. Results

The questionnaire was distributed to a total of 170 HCWs based on simple randomisation from the sampling frame received before the study commencement. A total of 161 respondents consented and completed the questionnaire. Therefore, the response rate was 90%. The prevalence of burnout among HCWs was 10.7% (17 response rates out of 161 participants), whereas 89.3% of participants were not experiencing burnout (Table 1). The majority of the respondents were under 40 years old (n = 111, 69.4%), female (n = 130, 81.8%), of Malay ethnicity (n = 141, 88.1%), married (n = 145, 90.6%), with less than 3 children (n = 92, 57.5%), and working as an MO (n = 49, 30.6%; Table 2).

Table 3 shows the results of the association analysis between independent variables and burnout symptoms among HCWs. All sociodemographic factors were not statistically significant. Three occupational factors showed significant association: having experienced verbal or physical aggression from patients (Χ^2^ = 6.002, *p* = 0.014), lower scores on relationships with colleagues (Χ^2^ = 8.008, *p* = 0.011), and lower scores on workplace efficiency (Χ^2^ = 7.378, *p* = 0.013).

Apart from that, 6 out of 7 EMR intrinsic factors showed significant association: time spent on documentation (Χ^2^ = 5.795, *p* = 0.023), the efficiency of screen layout and navigation (Χ^2^ = 11.445, *p* = 0.002), stability of the EMR system (Χ^2^ = 5.849, *p* = 0.016), integration of the EMR system with other electronic systems (Χ^2^ = 6.402, *p* = 0.011), having technical support (Χ^2^ = 6.443, *p* = 0.0011), and hardware and infrastructure (Χ^2^ = 9.705, *p* = 0.002). However, only a single EMR extrinsic factor showed a statistically significant bivariate analysis: overall satisfaction with the EMR system (Χ^2^ = 7.385, *p* = 0.013).

To determine the predictors for burnout symptoms among HCWs using the EMR, multiple logistic regression analysis was used. All 17 variables were tested, and analysis was performed using the forward method. Three significant predictors were obtained in this analysis: having experienced patients’ verbal and physical aggression, a lower score on relationships with colleagues, and a lower score for screen layout and navigation in the EMR system (Table 4). Respondents who had experienced verbal or physical aggression from patients were 5.7 times more likely to be experiencing burnout compared to those who never had experienced this. Respondents who scored lower in their relationships with colleagues were 3.9 times more likely to be experiencing burnout compared to those who scored higher. Respondents who scored lower in the efficiency of screen layout and navigation of the EMR system were 5.3 more likely to be experiencing burnout compared to participants who scored higher.

This model explained 26.1% of the variance of burnout (Nagelkerke R^2^ = 0.261), and it showed a good model fit (Hosmer Lameshow test *p* = 0.364). Based on the classification table, 88.1% of the data were classified correctly. The receiver operating characteristic showed a significant difference from this final model (*p* < 0.005), with an area under the curve of 0.829 (95% CI: 0.727–0.932). Thus, this final model could significantly discriminate in 82.9% of the cases.

## 4. Discussion

This is among the first studies in Malaysia using the single-item Mini Z survey as opposed to the MBI-HSS or CBI, which are more popular in measuring burnout among HCWs with other factors (occupational) than the EMR system. Overall, our study showed a lower prevalence, of 10.7%, compared to the burnout level among MOs (25.5%) in a tertiary hospital in Klang Valley in Malaysia [27]. Another study in Malaysia, conducted among house officers, MOs, and specialists (n = 313) in hospitals, reported that 15.9% had burnout symptoms [25]. Other studies using the Mini Z among HCWs have reported rates of 25–30% in the US and 25.6% in Canada [3,28,29]. The lower prevalence may be because the TPC-OHCIS system has been implemented since 2017, and users in the PHCs, especially the pilot site in Seremban, had already been familiarised with and accepted the system challenges. The EMR system may also have served as an aid in the PHC workflow setting. This is beneficial in terms of the staff management system at the PHC or Seremban District Health level. It prevents staff from encountering dissatisfaction that naturally leads to burnout. The difference in work in PHC settings compared to hospital settings may contribute to the lower prevalence of burnout in this study, especially the nature of work, the type of cases seen, the urgency of cases, and working hours in clinics.

In this study, respondents’ belief that they possessed poor relationships with their colleagues was significantly associated with burnout symptoms. Poor interpersonal relationships with colleagues remained significant after being included in the final model of multivariate analysis. This finding is similar to another study in Malaysia where conflict among colleagues became a significant predictor of burnout [30]. Interpersonal relationships are important in the workplace. Most HCWs spend more time at their workplace with their colleagues than at home with their families. Hence, those who are close and have good relationships with colleagues may find them to be a source of help when they encounter difficulties in operating the EMR system. As a result, a lower level of frustration towards the EMR system can improve burnout symptoms. Through good relationships with co-workers, HCWs can have a strong and cooperative working environment that fosters relaxation and serenity.

Among respondents, a belief that the screen layout and navigation were inefficient was a significant predictor for prevalent burnout symptoms. This is in line with a study performed in the US among primary care doctors, in which the inability to navigate the EMR system quickly was associated with high stress and burnout levels [20]. Doctors working in an academic psychiatric hospital also reported that too much ‘clicking’ was significantly associated with the negative perception of the EMR system and contributed to burnout [3].

The final predictor in our study was the history of exposure to physical or verbal abuse by clients or patients. In any circumstance, HCWs who have experienced physical or verbal abuse will likely experience a higher risk of burnout [31]. This also aligns with the Malaysian study of emotional exhaustion as a predictor of burnout when dealing with difficult patients [30]. In other studies, workplace violence has been essentially related to verbal abuse, which is more common than physical abuse [32,33]. A fast and simple online reporting system can be implemented to monitor HCWs who have experienced violence in the workplace so that immediate action can be taken. Appropriate counselling exercises or allowing unrecorded leave are a few actions that can help in managing their acute stress.

Even though only three predictors in the final model contributed to the risk of burnout symptoms among HCWs working with an EMR system, other factors are still worth mentioning. For example, a belief by respondents that their professional values were not aligned with department leaders was significantly associated with burnout symptoms. A survey among US primary care doctors found that professional value was a significant predictor of burnout using the Mini Z survey [20]. These findings were also similar to a study among Malaysian paediatricians that discovered that a lack of appreciation from superiors (*p* = 0.019) was a significant predictor of burnout [30]. With this information, we can plan activities with leaders and staff in the department to strengthen their relationships and suggest transparency in communication from time to time as one of the burnout intervention programs.

Apart from that, workplace efficiency could also affect burnout symptoms. This is supported by one local study in Malaysia that stated that a crowded workplace and a hostile working environment were significantly associated with burnout among HCWs [30]. The stability of the EMR system also contributed to low burnout symptoms. This is supported by findings in the US where EMR systems with a problem in their response time were associated with higher stress and burnout level [20]. Ensuring a stable internet connection and server at PHCs may prevent the increase in burnout symptoms among HCWs. Likewise, the availability of technical support could reflect the cause of low burnout symptoms. This is supported by a systematic review that reported that one of the barriers to the successful implementation of EMR systems was the lack of technical support, regarding hardware and software issues, to assist doctors [34]. Other studies have reported that limited hardware for the EMR system affected healthcare performance and user acceptance of the technologies [9,10]. However, this study is one of the first known to associate the EMR system hardware and infrastructure inadequacy with burnout among HCW.

### Strength and Limitation

The main strength of this study was that it used the latest EMR system (TPC-OHCIS) in primary healthcare settings. Therefore, the findings of this study can be used as a guide for system developers or system architects to focus on HCW preferences for EMR systems. Designing the correct layout that is preferred by the HCWs is very important because they are the end users of the EMR system in PHC clinics. Furthermore, the application’s user interface is culturally accepted, reducing confounding factors of system diversity. The random sampling technique of selecting participants is another strength of this study. Probability sampling would enable the result to be generalised widely.

This study has its limitations. The cross-sectional study design employed means that the temporal relationship between the outcome and the associated factors in the independent variables cannot be determined [35]. Additionally, an online survey was used instead of face-to-face interaction due to the COVID-19 pandemic and movement control orders from the government at the time. As a result, it is difficult to ascertain whether all the respondents truly comprehended the questions asked or if they simply answered the questions provided to them [36]. Finally, our study employing the use of a single-item questionnaire to measure burnout may not be truly a representation of the condition. The item is more skewed towards measuring emotional exhaustion, so the result should be interpreted with care.

## 5. Conclusions

To conclude, the use of an EMR system in PHCs was associated with a low level of burnout. The predictors of burnout symptoms among the users of the EMR system were the following: experiencing ineffective screen layout and navigation system, HCWs who had experienced physical or verbal abuse by patients, and having a poor relationship with colleagues. Hence, a continuous support system and empowerment program should be provided to all HCWs working with EMR systems. More efforts can be made to increase capacity-building and full utilisation of EMRs.

## Figures and Tables

**Table 1:** Prevalence of burnout.

No.	Variable	Frequency (n)	Percentage (%)
1.	0: I enjoy my work	106	66.3%
	1: I am stressed, but not burned out	37	23.1%
	2: I have burnout symptoms	15	9.4%
	3: Burnout will not go away	0	0.0%
	4: I feel completely burned out	2	1.3%
2.	E1. Burnout		
	- Yes (2–4)	17	10.7%
	- No (0–1)	143	89.3%

**Table 2:** Respondents’ characteristics.

No.	Variable Sociodemographic	Frequency (n)	Percentage (%)
1.	Age		
	Less than 40	111	69.4%
	40 or more	49	30.6%
2.	Gender		
	Male	30	18.8%
	Female	130	81.8%
3.	Ethnicity		
	Malay	141	88.1%
	Indian	12	7.5%
	Chinese	7	4.4%
4.	Marital status		
	Single	12	7.5%
	Married	145	90.6%
	Divorced	2	1.3%
	Widow/widower	1	0.6%
5.	Number of children		
	Less than 3	92	57.5%
	3 or more	68	42.5%
6.	Job designation		
	Family medicine specialist	8	5.0%
	Medical officer	29	30.6%
	Assistant medical officer	22	13.8%
	Nurse	81	50.6%

**Table 3:** Result of bivariate analysis.

No.	Variable Sociodemographic	Burnout Symptom Yes n (%)	Burnout Symptom No n (%)	X^2^	df	*p*-Value
1.	Age			0.45	1	0.502
	<40 years	13 (11.7%)	98 (88.3%)			
	>40 years	4 (8.2%)	45 (91.8%)			
2.	Gender			1.42	1	0.320 ^a^
	Male	5 (16.7%)	25 (83.3%)			
	Female	12 (9.2%)	118 (90.8%)			
3.	Ethnicity			3.80	2	0.150
	Malay	15 (10.6%)	126 (89.4%)			
	Indian	0 (0.0%)	12 (100%)			
	Chinese	2 (28.6%)	5 (71.4%)			
4.	Marital status			1.97		0.372 ^a^
	Single/divorced/widow	0 (0.0%)	15 (100%)			
	Married	17 (11.7%)	128 (88.3%)			
5.	Number of Children			1.33	1	0.248
	<3	12 (13.0%)	80 (87.0%)			
	>3	5 (7.4%)	63 (92.6%)			
6.	Job designation			4.98		0.173 ^a^
	FMS	0 (0.0%)	8 (100.0%)			
	MO	9 (18.4%)	40 (81.6%)			
	AMO	2 (9.1%)	20 (90.9%)			
	Nurse	7 (10.6%)	75 (92.6%)			
7.	B1. Years of working experience			2.487	1	0.115
	Less than 10 years	9 (15.8%)	48 (50.9%)			
	10 years or more	8 (7.8%)	95 (92.2%)			
8.	Working hours per week			2.874	1	0.131 ^a^
	Less than 40 h	0 (0.0%)	21 (100%)			
	40 h or more	17 (12.2%)	122 (87.8%)			
9.	Number of patients seen per day			1.589	1	0.207
	Less than 30	5 (7.1%)	65 (92.9%)			
	30 or more	12 (13.3%)	78 (86.7%)			
10.	Patients’ verbal or physical aggression			6.002	1	0.014 *
	Yes	14 (16.1%)	73 (77.8%)			
	No	3 (4.1%)	70 (65.2%)			
11.	Professional value aligned with the department leader			2.781	1	0.111 ^a^
	Lower score (disagree)	6 (18.8%)	26 (81.3%)			
	Higher score (agree)	11 (8.6%)	117 (91.4%)			
12.	Relationship with colleagues			8.008	1	0.011 ^a^*
	- Lower score (bad)	7 (25.9%)	20 (74.1%)			
	- Higher score (good)	10 (7.5%)	123 (92.5%)			
13.	Workplace efficiency			7.378	1	0.013 ^a^*
	- Lower score (bad)	11 (19.6%)	45 (80.4%)			
	- Higher score (good)	6 (5.8%)	98 (94.2%)			
14.	Time spent on documentation.			5.795	1	0.023 ^a^*
	- Lower score (bad)	9 (20.0%)	36 (80.0%)			
	- Higher score (good)	8 (7.0%)	107 (93.0%)			
15.	The efficiency of screen layout and navigation.			11.445	1	0.002 ^a^*
	- Lower score (bad)	11 (23.4%)	36 (76.6%)			
	- Higher score (good)	6 (5.3%)	107 (94.7%)			
16.	Stability of the system			5.849	1	0.016 *
	- Lower score (bad)	12 (17.4%)	57 (82.6%)			
	- Higher score (good)	5 (5.5%)	86 (94.5%)			
17.	Integration			6.402	1	0.011 *
	- Lower score (bad)	13 (17.1%)	63 (82.9%)			
	- Higher score (good)	4 (4.8%)	80 (95.2%)			
18.	Data privacy and security			3.690	1	0.055
	- Lower score (bad)	10 (16.7%)	50 (83.3%)			
	- Higher score (good)	7 (7.0%)	93 (93.0%)			
19.	- Technical support			6.443	1	0.011 *
	- Lower score (bad)	12 (17.9%)	55 (82.1%)			
	- Higher score (good)	5 (5.4%)	88 (94.6%)			
20.	Hardware and infrastructure			9.705	1	0.002 *
	Lower score (bad)	12 (20.7%)	46 (79.3%)			
	Higher score (good)	5 (4.9%)	97 (95.1%)			
21.	Duration of using EMR system			0.710	1	0.330 ^a^
	- 6 months or less	2 (18.2%)	9 (81.8%)			
	- More than 6 months	15 (10.1%)	134 (89.9%)			
22.	Consistent training is needed			2.742	1	0.148 ^a^
	- Lower score (disagree)	5 (20.0%)	20 (80.0%)			
	- Higher score (agree)	12 (8.9%)	123 (91.1%)			
23.	Good proficiency in computer skills			0.128	1	0.663 ^a^
	- Lower score (disagree)	2 (13.3%)	13 (86.7%)			
	- Higher Score (agree)	15 (10.3%)	130 (89.7%)			
24.	Helps with patients’ communication			3.190	1	0.127 ^a^
	- Lower score (disagree)	7 (18.4%)	31 (81.6%)			
	- Higher score (agree)	10 (8.2%)	112 (91.8%)			
25.	Adherence to guidelines and reduces medical error			0.148	1	0.749 ^a^
	- Lower score (disagree)	4 (12.5%)	28 (87.5%)			
	- Higher score (agree)	13 (10.2%)	115 (89.8%)			
26.	Overall satisfaction			7.385	1	0.013 ^a^*
	- Lower score (disagree)	7 (25.0%)	21 (75.0%)			
	- Higher score (agree)	10 (7.6%)	122 (92.4%)			

^a^ Fisher’s exact test; * variable included in multivariable analysis.

**Table 4:** Result of multivariate analysis.

Variable	β	SE	Wald	aOR	Lower 95% CI	Upper 95% CI	*p*-Value
Patient’s verbal or physical aggression (yes)	1.736	0.708	6.018	5.676	1.418	22.727	0.014 *
Relationship with colleagues (lower score)	1.360	0.604	5.073	3.898	1.193	12.733	0.024 *
Efficiency on screen layout and navigation (lower score)	1.659	0.575	8.314	5.256	1.701	16.237	0.004 *

* *p* value < 0.05.

## Data Availability

The dataset analysed in this study is not available publicly due to local regulation imposed by the Medical Review and Ethics Committee (MREC), Ministry of Health Malaysia. Data could be obtained via written permission to the State Health Department of Negeri Sembilan and the Director General of Health, Ministry of Health Malaysia. Data request may be sent to Seremban District Health Office, 70300 Seremban, Negeri Sembilan, Malaysia or emailed directly to the corresponding author.
